# Non-halogenated Ionic Liquid Dramatically Enhances Tribological Performance of Biodegradable Oils

**DOI:** 10.3389/fchem.2019.00098

**Published:** 2019-02-28

**Authors:** Patrick Rohlmann, Bulat Munavirov, István Furó, Oleg Antzutkin, Mark William Rutland, Sergei Glavatskih

**Affiliations:** ^1^Machine Design, KTH Royal Institute of Technology, Stockholm, Sweden; ^2^Department of Chemistry, KTH Royal Institute of Technology, Stockholm, Sweden; ^3^Chemistry of Interfaces, Luleå University of Technology, Luleå, Sweden; ^4^Surfaces, Processes and Formulation, RISE Research Institutes of Sweden, Stockholm, Sweden; ^5^Department of Electrical Energy, Metals, Mechanical Constructions and Systems, Ghent University, Ghent, Belgium

**Keywords:** biodegradable oil, ionic liquid, wear, friction, boundary lubrication, NMR

## Abstract

It is demonstrated that a phosphonium orthoborate ionic liquid may serve as a wear reducing additive in biodegradable oils at steel-steel surfaces in the boundary lubrication regime. Tribological tests were performed in a ball-on-three plate configuration. A set of surface characterization techniques—SEM/EDS, FIB and white light interferometry were used to characterize surfaces following the tribotests and to observe the formation of any tribofilms. ^11^B NMR was used to follow changes in the composition of the ionic-liquid-oil blends and to identify boron-containing decomposition products after the tribotests. The ionic liquid reduces the wear of steel surfaces by up to 92% compared to the neat oil at 90°C; it is shown that the reduction in wear can be correlated with the formation of boron enriched patches in the boundary films.

## Introduction

The mounting pressure for a transition from mineral to biodegradable oils in many industrial applications implies a number of technological challenges. One of them is to identify the next generation of wear reducing additives. Most current anti-wear additives, such as zinc dialkyldithiophosphates (ZDDP) and tricresyl phosphates (TCP), were developed in late 1940s for use with mineral oils. Despite their good wear reducing properties for lubricated steel-steel contacts (Martin, 1999; Fujita and Spikes, 2004), there are environmental concerns regarding the use of ZDDP and TCP. ZDDP containing formulations tend to form zinc and phosphorus rich ashes in internal combustion engines, which poison catalysts and consequently worsen the exhaust quality (Xie et al., 2016). Similar problems are related to the use of TCP, which promotes formation of toxic species upon its decomposition at lubrication contacts at elevated temperatures (Michaelis, 2011; Ramsden, 2013), and it is intrinsically toxic in its pure form (Goldstein et al., 1988). Moreover, the wear reducing performance of these additives in biodegradable oils is rather moderate according to four-ball configuration mechanical tests for steel-steel contacts: it has been reported that vegetable oils with ZDDP additives result in only up to 13% reduction of the wear scar diameter when compared with the neat oil (Mahipal et al., 2014). Choi et al. have studied the performance of TCP in vegetable oils and reported up to 30% reduction in the wear scar diameters when compared to the neat oils (Choi et al., 1997).

A range of alternative additive approaches have been examined, including other phosphorus compounds (Johnson and Hils, 2013) and nanoparticles (Shahnazar et al., 2016). Hybrid approaches have also been used, for example when combining diamond nanoparticles with phosphoesters (Acharya et al., 2018). Another promising avenue to address the challenge is the use of ionic liquids (ILs). Knowledge of ILs in lubrication is rapidly expanding and the growing number of publications on ILs as neat lubricants or additives to oils (Zhou and Qu, 2017) is clear evidence of this. Ionic liquids also provide the opportunity to control the friction properties via the use of electric fields due to their ionic nature (Sweeney et al., 2012; Cooper et al., 2016)—so called tribotronics (Glavatskih and Höglund, 2008). However, a significant fraction of the published work is on halogenated ILs, which for historical reasons are commercially available, but are of limited interest to industry (Totten et al., 2003) due to environmental and health reasons. The “rear-view mirror” approach to the development of the ionic liquids field in general—that is to say the vicious circle of ionic liquid selection based on previous studies for benchmarking purposes—was highlighted at the recent Faraday Discussions on Ionic Liquids, and indeed formed the basis of the closing address (Jessop, 2018). A further key element concerning the “sustainability” of ionic liquids was a salutary reminder that one cannot consider the relative merits of ILs vs. their conventional counterparts in their application alone, but that the environmental impact of their manufacture should also be compared. Successful candidates for industrial applications in the field of tribology (the study of friction, lubrication, and wear) need to be non-halogenated ILs engineered to meet the requirements of the lubrication industry (Shah et al., 2011). The choice and structure of cations and anions are dictated by the application specifics such as the materials to be lubricated, the contact geometry and pressure, as well as the speed, ambient conditions and type of movement (sliding/rolling, reciprocating/continuous). An example of an exception to the use of halogenated ILs is the work of Kronberger et al. They investigated tribological performance of a number of neat ILs and IL solutions in glycerol (Kronberger et al., 2012) using sulfur based anions [sulfates (Pejakovic et al., 2012) and sulphonates] and nitrogen based cations (n-alkylammonium, cholines, pyrrolidinium). In some cases both friction and wear were reduced compared to neat glycerol, with neat ILs and solutions performing similarly. XPS analysis of the worn surfaces suggest that the performance is enhanced by formation of iron sulfide tribolayers on the metal surfaces. The same authors have also pointed out that miscibility of an IL with a carrier fluid is crucial for their deployment (Kronberger et al., 2014).

Xie et al. have recently compared ZDDP with phosphonium based ionic liquids (ILs) and concluded that ILs have less impact on the functionality of the catalysts (Xie et al., 2016). Phosphonium cations are known for their wear reducing performance and the anti-wear boundary films produced on the lubricated surfaces have been shown to contain phosphorus (Westerholt et al., 2015). A corrosion inhibition effect of phosphonium cations has also been observed (Kondo et al., 2012). It has also been reported that ILs containing phosphorus in both anion and cation are efficient anti-wear additives to non-polar oils (Qu et al., 2015). An enhancement in the anti-wear performance of phosphonium ILs as additives to a mineral oil was achieved by adding a borate ester (Sharma et al., 2016) to the oil blend. Boron is known to form friction and wear reducing compounds in lubricated contacts (Shah et al., 2013) and ILs consisting of phosphorus and boron based ions have thus been the target of synthesis (Shah et al., 2011). Such ILs have formed the focus of a range of nanotribology and model studies (Li et al., 2016; Cooper et al., 2017; Cowie et al., 2017) confirming this promise. In this work, boron and phosphorus are incorporated in a single IL, as the anion and cation respectively, trihexyltetradecylphosphonium bis(mandelato)borate (P-BMB), which is then used as an additive to biodegradable oils. The goal is to study how the IL additive improves oil lubrication performance on steel surfaces, and to relate this to the additive properties, and to any antiwear films generated on the surfaces.

## Materials and Methods

**Figure 1:**
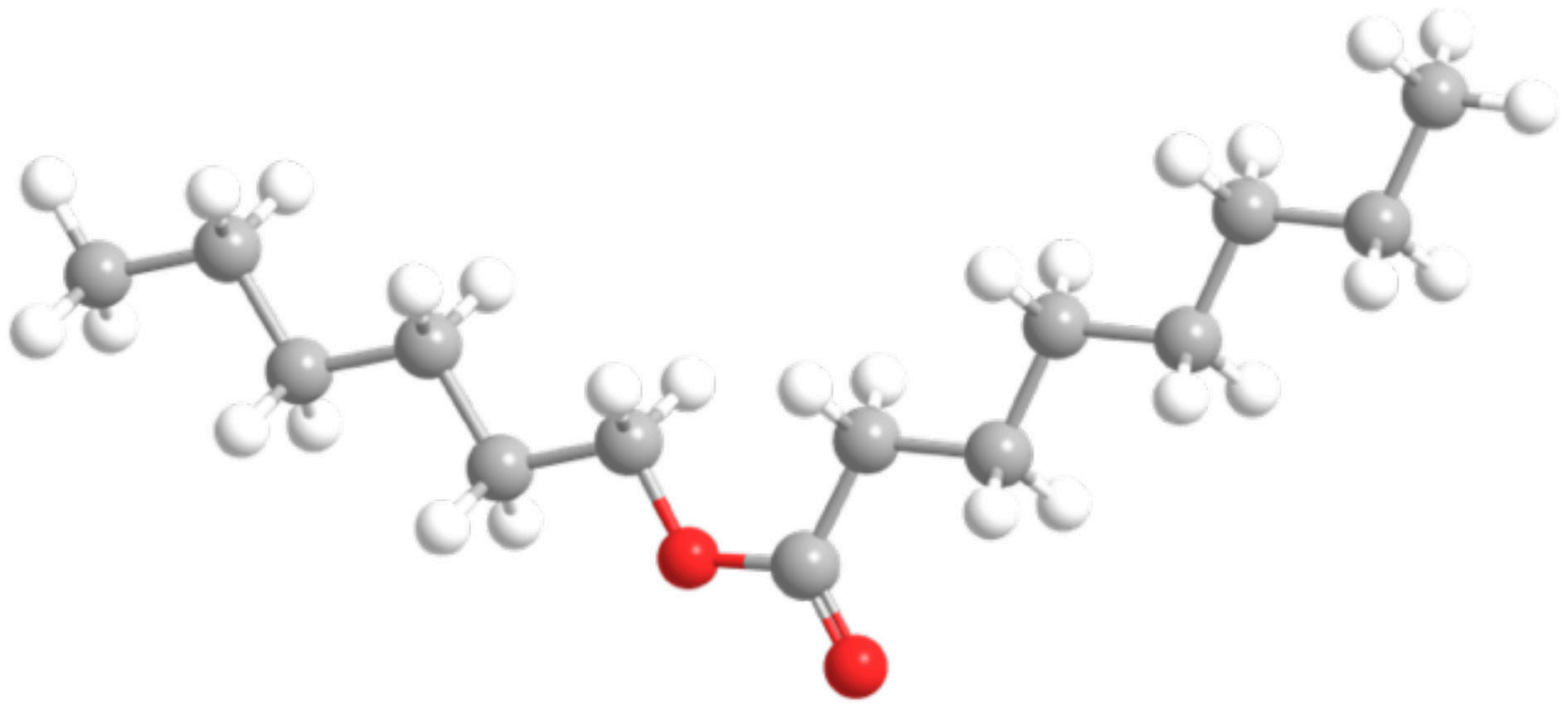
The structure of the C8-C6 monoester, hexyl-octanoate, denoted in the text as ME. Red, O; gray, C; white, H.

P-BMB was synthesized and characterized as previously described (Shah et al., 2011). The higher polarity of biodegradable oils compared to conventional mineral oils increases their affinity for the surface and may also have implications for the transport properties of the IL. Two representative biodegradable oils were selected for the tests. Oil soluble polyalkylene glycol (OPAG), UCON OSP46 (Dow Chemicals inherently biodegradable; more than 35 % during 28 days), and a monoester (ME), Estisol 240 (Estichem, readily biodegradable; more than 60% during 28 days), were used in the lubrication tests. The exact chemical structure of OPAG is undisclosed which underlines some of the challenges of performing relevant tribological research from a chemical perspective. Estisol 240 is a C8-C6 monoester, hexyl-octanoate with a structure shown in Figure 1 and has a non-polarity index of 32. To ensure homogeneous blends, the blends were ultrasonicated for at least 60 min at 50°C. Table S1 shows viscosities of IL, oils and the blends of oils with IL. Viscosities were measured with a HR-2 rheometer, TA Instruments.

### Solubility Tests of Ionic Liquids

Several IL-oil mixtures were prepared with IL concentrations varied within 0–20 wt.%. After having been thoroughly mixed, the samples were centrifuged at a speed of 6,500 rpm. An equal amount of supernatant was then carefully taken from all mixtures, centrifuged and placed into separate NMR tubes. Quantitative ^31^P and ^1^H NMR were run on the resultant samples of the ME and OPAG blends. Obtained NMR spectra were processed: “0” and “1” order phase corrections were applied; the baseline was corrected using a first order polynomial correction. After that spectral regions corresponding to the IL liquid were integrated. The resultant integral values were normalized according to the integral value obtained from the sample with an IL concentration of 1 wt%. The initial concentrations were determined from the actual IL and oil weights used before the centrifugation.

### Test Procedure

The same rheometer that was used for measuring lubricant viscosities was utilized for the lubrication tests. A set-up with a ball-on-three-plates configuration as shown in Figure S1 was installed. (This setup allows high contact pressures, continuous sliding—typical for lubricated contacts, faster lubricant consumption and the plates are compatible with SEM analysis). The ball was pressed against the plates with an initial maximum Hertzian contact pressure of 1.11 GPa. This load, corresponding to 21 N per contact, was selected to place the contact in the boundary regime, for which this is an appropriate pressure; similar contact pressures have been used in related publications. 1.13 GPa was employed to study neat IL lubrication of steel (Garcia et al., 2014), while 1.0 GPa maximum pressure was used in two studies of IL as additives to glycerol (Kronberger et al., 2012; Pejakovic et al., 2012; Garcia et al., 2014). Each test was run with a sliding speed of 0.2 ms^−1^ for a sliding distance of 2000 m. (This sliding speed and distance provide a balance between experimental time and a suitable degree of wear). Normal and friction forces were recorded with an acquisition frequency of 1 Hz. Friction coefficients during 500 m of sliding were then averaged. Oil bath temperature, set to 90°C, was regulated by a Peltier element. A test run required 1.2 ml of lubricant and was repeated 4 times. Relative humidity, measured during the tests, was 40 ± 5% at an ambient temperature of 22 ± 2°C. The ⊘12.7 mm steel balls (AISI 52100, Rotera Kullager AB, Täby, Sweden), used in the lubrication tests, had a measured roughness of R_a_ = 0.074 ± 0.006 μm and a hardness of 60–67 HRC (Rockwell Hardness, according to the supplier). The mating surface, AISI 52100 steel plates, had a measured hardness of 64 HRC and roughness of R_a_ = 0.142 ± 0.028 μm. The roughness was measured with a Taylor Hobsen PGI 800 Profilometer from Talysurf. The hardness was determined by measuring indents with an optical microscope from Nikon (MM-60/L3). The plates were cut from the rolling bearing discs and had the following dimensions: 3 mm in thickness, 6 mm in width and 7 mm in length. Before each test, balls and plates were cleaned in several steps. First, they were ultrasonicated in acetone for 30 min and then dried under a dry air stream. Next, they were ultrasonicated in isopropanol for 30 min and finally dried by dry air stream again. After the tribological experiments balls and plates were rinsed with ethanol and ultrasonicated twice (30 min each time), using fresh heptane each time. Then they were dried under a dry air flow and placed in a vacuum oven for 3 days at 50°C.

### Wear, Interfacial Film, and Oil Analysis

The wear volume of the plates was measured by using a contact profilometer (Taylor Hobson PGI800) and an optical profiler (Zygo7300). The average wear volume *W*_*v*_ was calculated for each set of three plates and used to obtain the wear coefficient:

(1) $$\begin{array}{l} k=W_{v}/\left(F_{N}\times s\right), \end{array}$$

where *F*_*N*_ is the normal contact force on a single plate and *s* is sliding distance (Archard, 1953).

A Scanning Electron Microscope (SEM), JEOL 7800F, was employed to take images of the wear tracks at a beam energy of 10 kV. Distributions of chemical elements for selected points inside and outside the wear tracks were obtained by using an Energy Dispersive X-ray Spectroscopy (EDS), Bruker Quantax system.

A focused ion beam (FIB-SEM FEI NOVA 600) was used to create cross section cuts through the boundary film. Prior to each cut EDS spectra were recorded with 10 *kV*, followed by a deposition of a thin layer (~500 *nm*) of platinum on top of the investigated area (100 *pA* current, 30 *kV* voltage). Thereafter, the focused ion beam with a beam current of 3 *nA* and 30 *kV* voltage was utilized to create a wedge cut-out with a maximum depth of 4 μ*m* at the edge of cross section. SEM images of the cut-outs were recorded with a 5 *keV* beam voltage.

^11^B NMR spectra of the oil blends before and after the lubrication tests were recorded at 160.5 MHz on a Bruker Avance III NMR spectrometer. A standard Broad Band probe from Bruker was used. Spectral width was set to 64 kHz and all NMR measurements were performed at 60°C. A 50 Hz exponential filter was applied to all spectra to reduce the noise level.

## Results and Discussion

**Figure 2:**
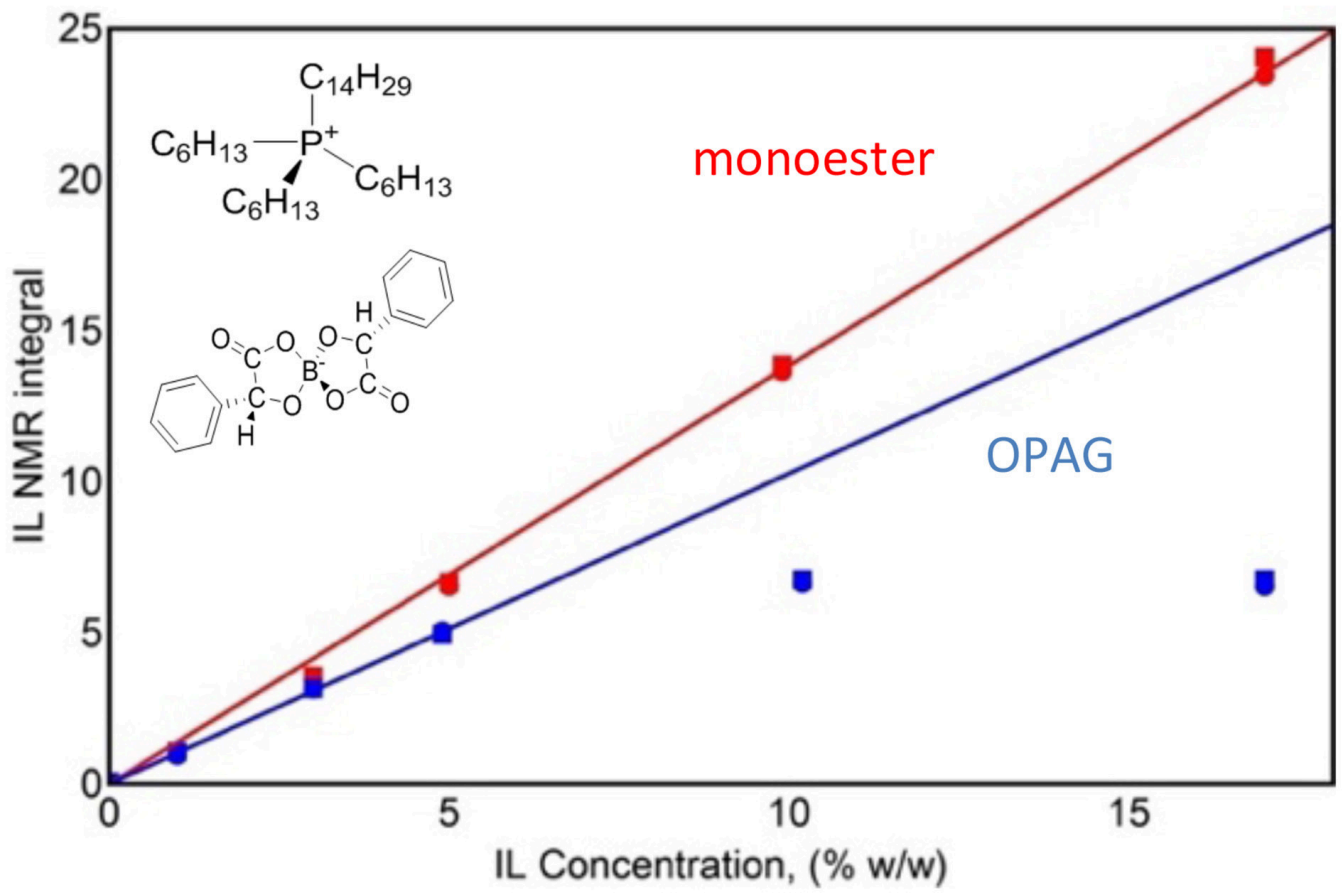
IL NMR integral value for supernatants of centrifuged mixtures as a function of the initial concentration. Circles are for ^31^P NMR and squares for ^1^H NMR. Solid lines are linear fits to the low-concentration experimental data.

The normalized spectral integral values obtained for the blends prepared at room temperature are shown in Figure 2. The IL NMR integral values (corresponding to the amount of IL in the supernatants) for the ME blends are proportional to the initial concentrations of IL in a mixture. For the OPAG blends, a plateau of the NMR integral values is observed for the initial concentrations higher than 5 wt%. Addition of IL above this concentration to OPAG resulted in the precipitation of the added IL during the centrifugation stage. It is thus concluded that the IL is soluble in ME within the concentration range studied, whereas it has a limited solubility (up to 5 wt% at RT) in OPAG.

The self-diffusion coefficients of the ions of the IL in oils were approximately one order of magnitude higher relative to their values in neat IL. Thus, the distribution of IL in the ME is considered stable and homogeneous and no liquid droplets exist.

**Figure 3:**
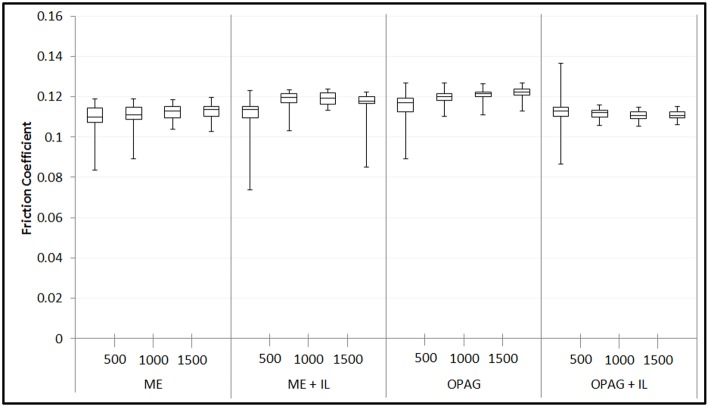
Average friction coefficients over a sliding distance of 2,000 m. The data is divided into four intervals of the sliding distance to allow any distance or time dependent phenomena to be observed.

Blends of 5 wt% IL with ME and OPAG were thus used for the lubrication tests (the concentrations of phosphorus and boron in the blends were 1,948 and 680 ppm, respectively). The results for all samples are shown in Figure 3. In the beginning of each test the running-in process resulted in a somewhat larger variation of the coefficient of friction. This variation decreased with time in all cases. However, for ME + IL more unstable friction returned toward the end of the test. Particularly after running-in it is clear that the OPAG+IL friction coefficients are uniformly lower than for OPAG, by ~10% The full data sets are shown in Figure S2. The fact that the friction coefficient values are similar, despite a 6-fold difference in viscosity between ME and OPAG samples at 90°C strongly indicates that the contact indeed operates in the targeted boundary lubrication regime, as opposed to “full film lubrication,” whereby a liquid film separates the surfaces. Since hydrodynamic effects are insignificant in boundary lubrication, it is concluded that surface protection is achieved through the interactions of oil molecules and additives with the lubricated surfaces.

**Figure 4:**
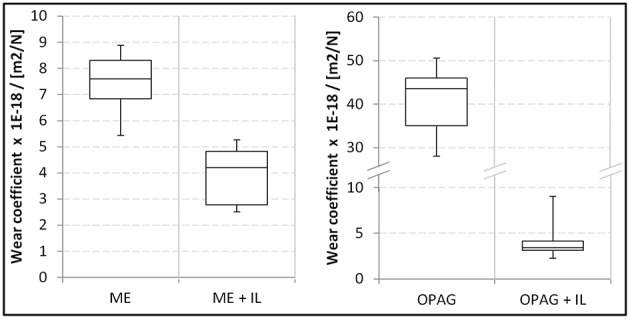
Wear coefficients for the plates lubricated by ME, OPAG and their blends with IL. Note that the axis for OPAG has been broken since the wear for the neat oil was much larger.

**Figure 5:**
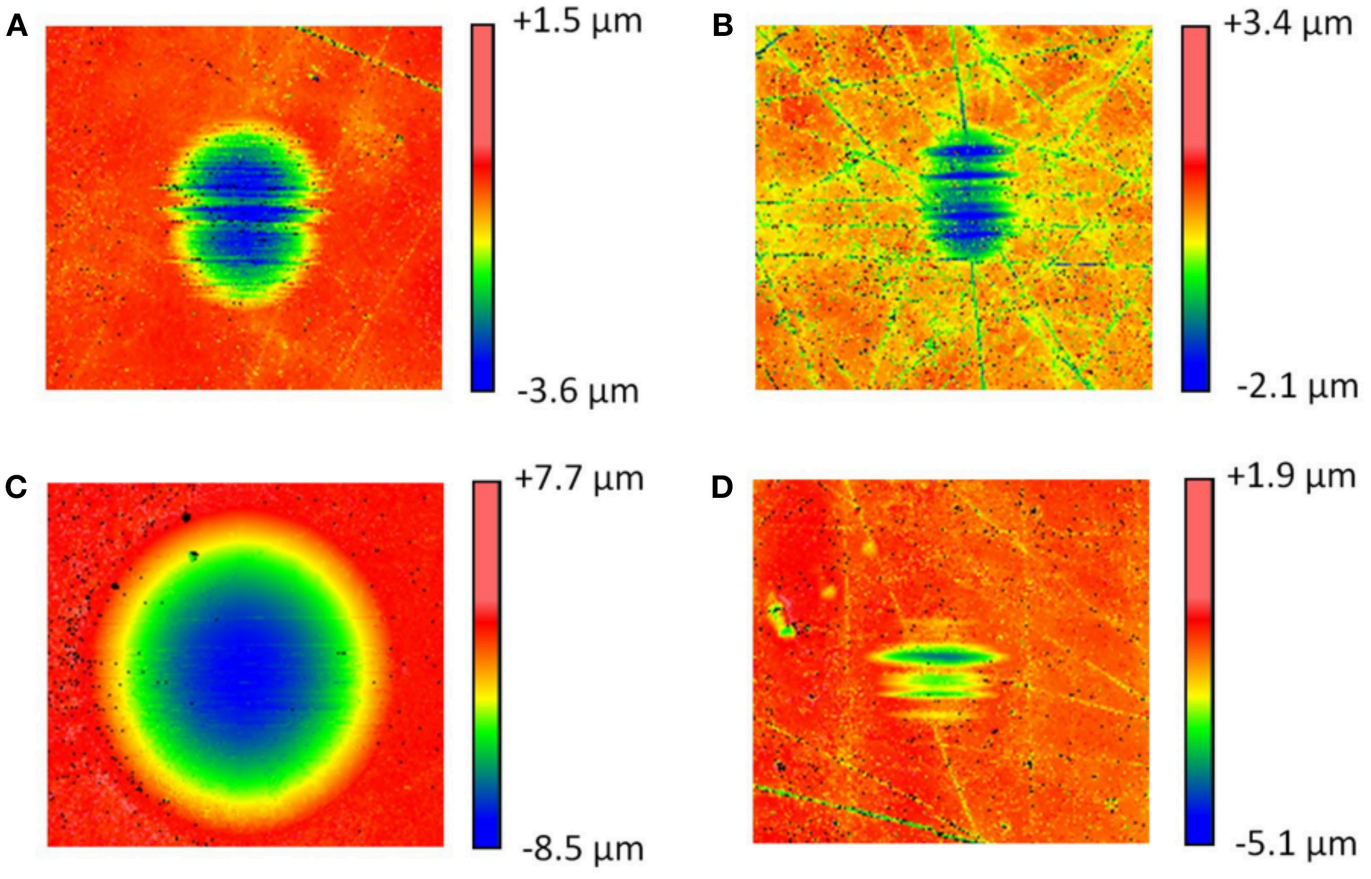
Optical images (1 × 1 mm) of the wear scars for **(A)** ME, **(B)** ME + IL, **(C)** OPAG, **(D)** OPAG + IL.

Monoesters are recognized as surface active compounds and are often used as friction modifiers with an anti-wear functionality (Spikes, 2015). Consequently, in these experiments neat ME provided ~5 times lower wear compared to OPAG. Similar trends for ME and polyglycols have previously been reported (Chen et al., 2009). The wear coefficients are presented in Figure 4. It can be seen that addition of IL resulted in significantly lower wear. The reduction was much larger (92%) for the OPAG/IL blend, reflecting the fact that OPAG has poorer intrinsic wear protection than ME. Optical images of the wear scars are shown in Figure 5. The wear scars attained with the neat oils are deep and are of a non-circular shape (see the ESI for details). Compared to the neat oils, the blends with IL produced much smaller and shallower wear scars. Scratches from the manufacturing process (polishing) were still visible on the plate surfaces after the tests. The depth of the scratches was similar to the wear scar depth for the oil/IL blends, emphasizing the mildness of the wear.

**Figure 6:**
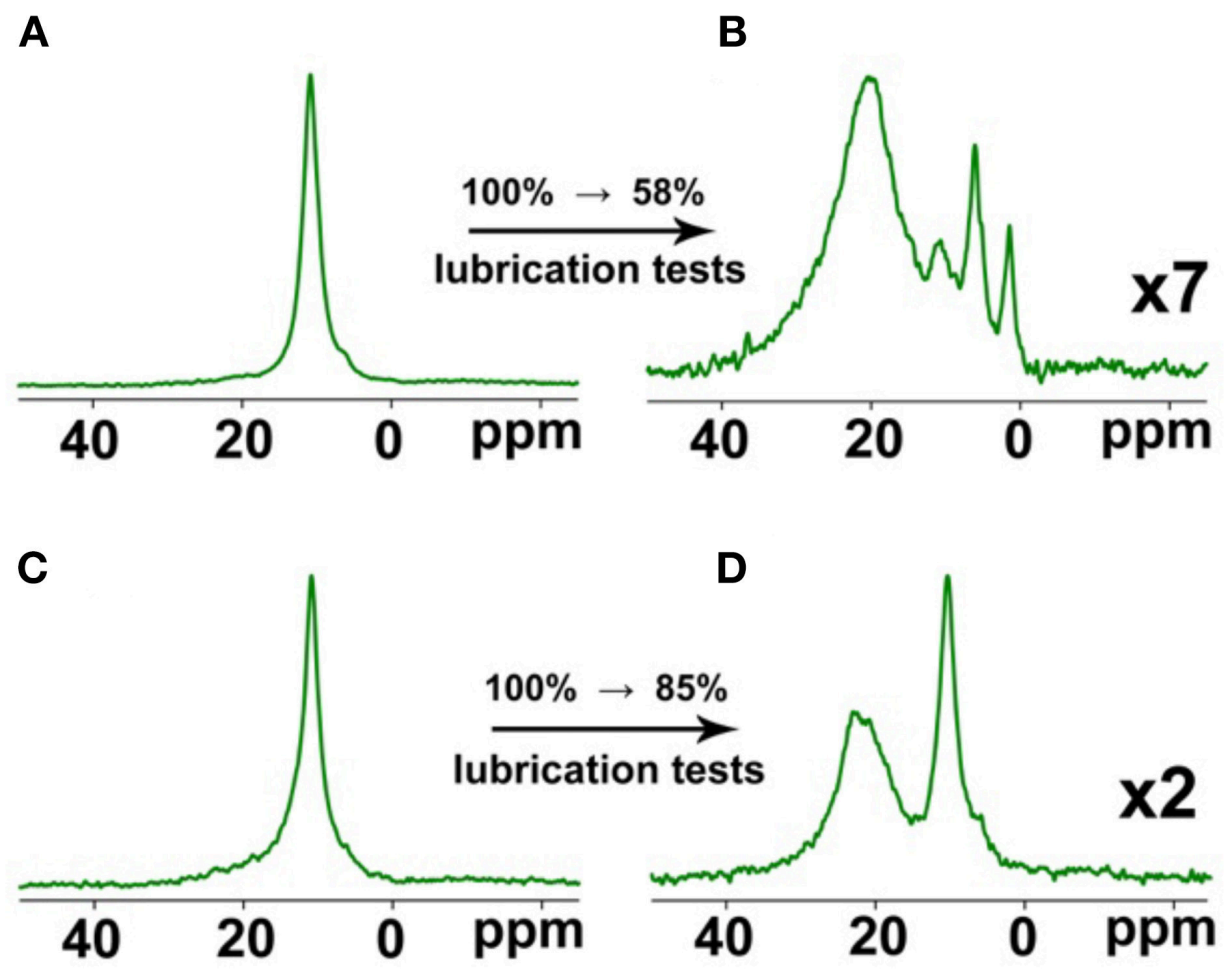
^11^B NMR spectra and changes in the relative values of the spectral integral intensities for the ME + IL **(A,B)** and OPAG + IL (**C,D** blends). Intensities of the spectra recorded after the lubrication tests are amplified.

Biodegradable oils are designed to degrade if they leak into the environment but to remain stable in the lubrication system. However, due to high thermo-mechanical stresses in the lubricated interface, the oil *may* degrade in the contact producing compounds that can also be reactive toward ions, thus accelerating consumption of the IL and shortening lubricant service life. Note that reactivity can also have a positive effect if it contributes to the formation of stable, so called “antiwear” films on the surfaces in contact. Consumption of the IL in the blends was studied with NMR spectroscopy. The ^11^B NMR spectra of ME/IL and OPAG/IL blends taken before and after the lubrication tests were compared. The spectra before the tests showed a single resonance line, Figure 6, corresponding to the neat IL but this changed after the tests. Additional spectral lines appeared, indicating the orthoborate anion reacting to yield boron in a new chemical environment. The overall signal intensity (obtained by integration between −100 and 100 ppm) also decreased, relative to the initial (before the lubrication tests) situation. Those relative values are shown in percent in Figure 6. This finding indicates a depletion of soluble boron-containing (including the original orthoborate anions) species from the oil blends during the lubrication tests.

This effect is particularly strong for the ME blend where, in addition, hardly any of the boron-containing solutes are in the form of the original orthoborate species (as indicated by the near disappearance of the original resonance line at ca. 11 ppm). For the OPAG blend, neither the depletion nor the chemical reaction is as pronounced. Since the amount of boron in the oil blends decreased during the lubrication tests, it can be assumed that boron accumulated in the boundary films.

**Figure 7:**
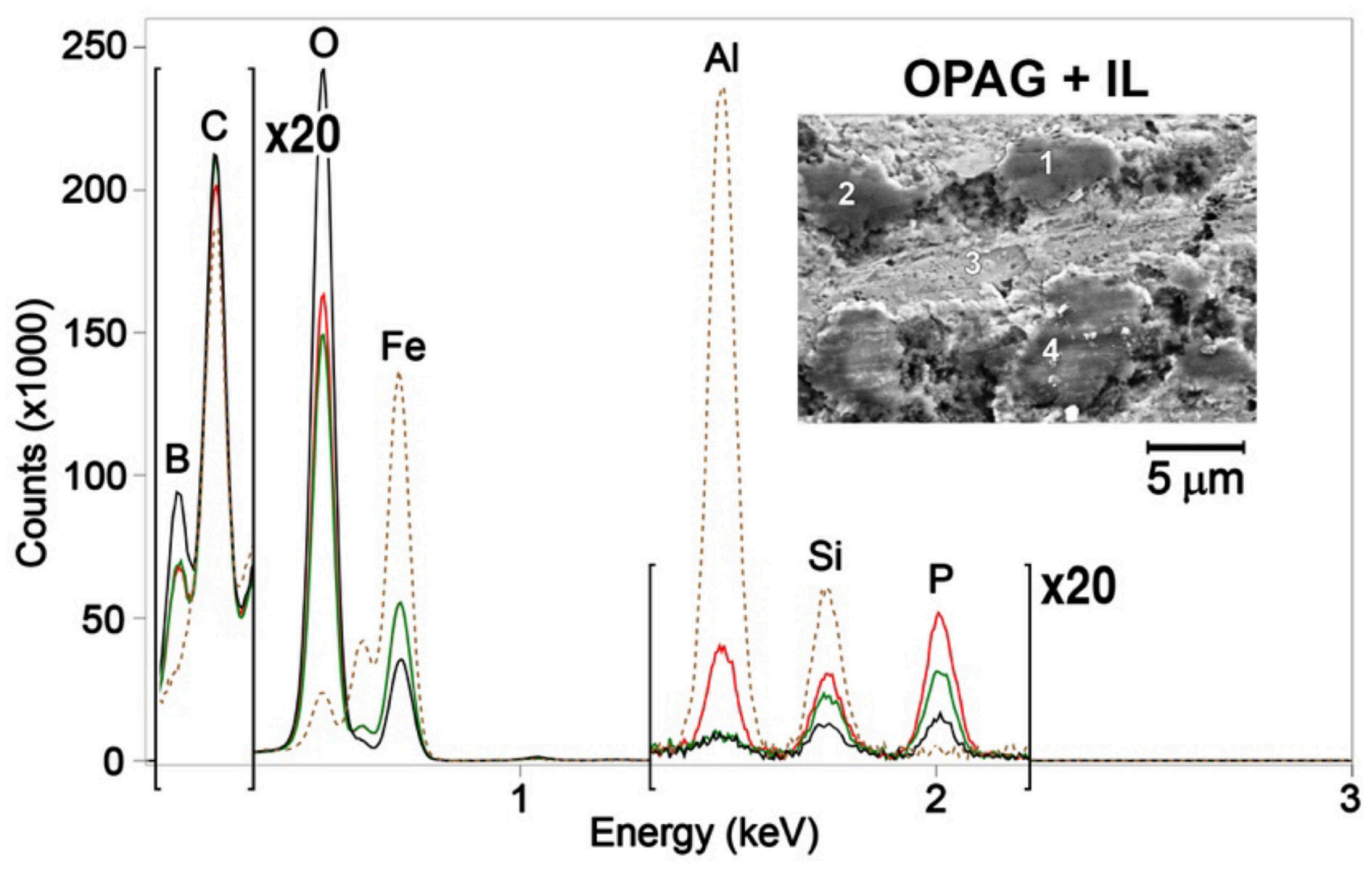
EDS spectra for ME/IL obtained at several points as shown in the inserts. Spectra color codes are for different points: 1—red, 2—blue, 3—green, 4—black. The brown dashed line is for the unworn surface.

**Figure 8:**
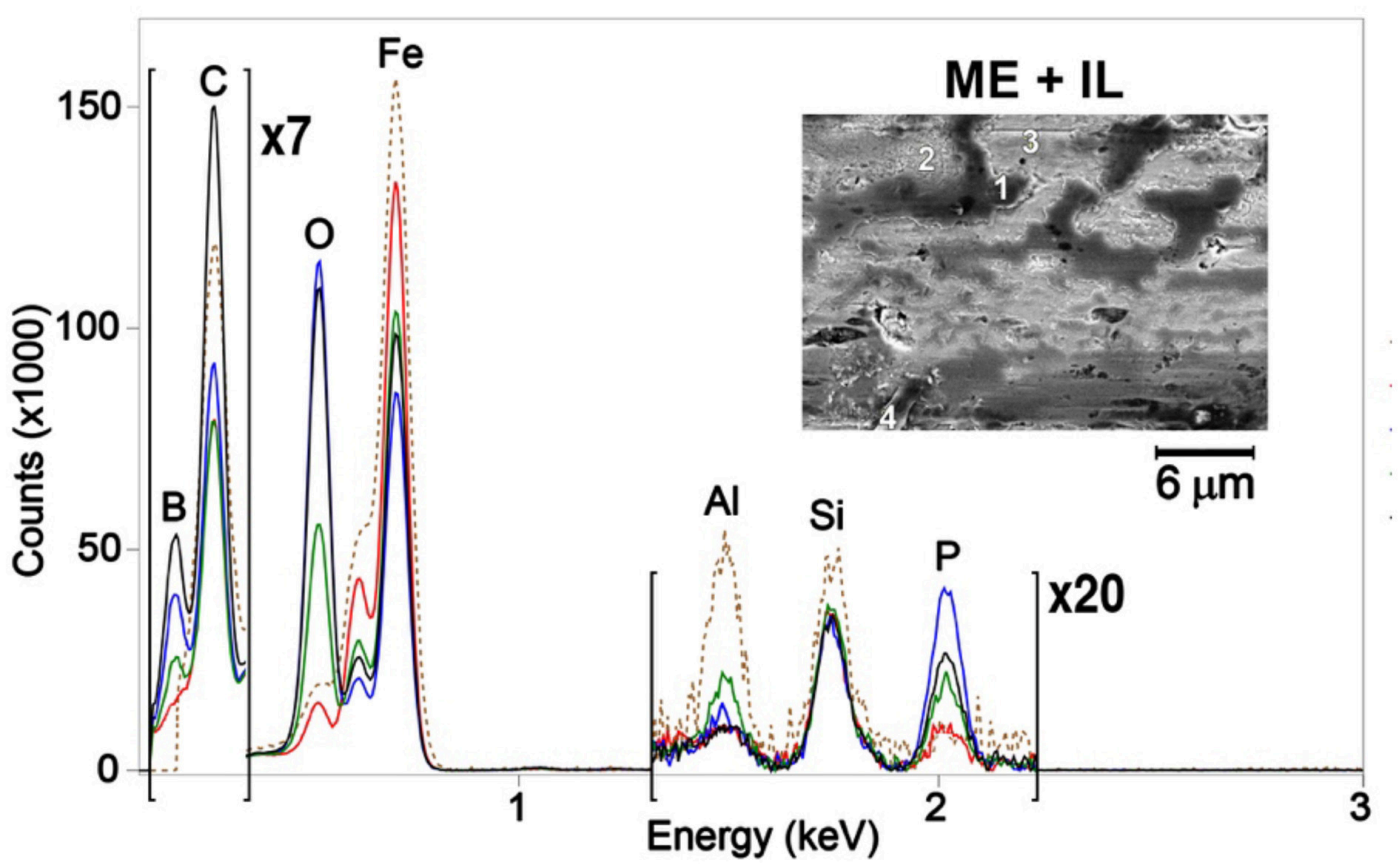
EDS spectra for OPAG/IL obtained at several points as shown in the inserts. Spectra color codes are for different points: 1—red, 2—blue, 3—green, 4—black. The brown dashed line is for the unworn surface.

To investigate the veracity of this assumption, both the unworn and worn steel surfaces were analyzed using SEM-EDS. SEM images (insets in Figures 7, 8) revealed a patchy boundary film structure inside the wear track for both oil blends. The patches appeared to be larger in the case of OPAG/IL compared to the ME/IL blend. EDS spectra were recorded at a number of points located within the wear scar to probe the elemental composition of the patches. Reference EDS spectra were recorded at points located on the unworn surface outside the wear scar. Point positions are marked by numbers in the insets in Figures 7, 8. Spectra for the balls are not shown as no phosphorus or boron were detected in the wear scars on the ball surfaces. This is reasonable considering the less severe tribological conditions experienced by the ball surface due to the shorter contact time (see Figure S1).

The unworn plate surface contained iron, carbon, oxygen and traces of aluminum. Aluminum originates from the grinding and polishing process. Almost no aluminum was detected inside the wear scar, indicating that it was located within the very top surface layer and was removed by rubbing during the lubrication tests. The worn surfaces also showed an increased amount of oxygen and a decreased amount of iron. The lower intensity of the iron peak indicates that the boundary film was formed on top of the metal surface during the rubbing process. EDS spectra for a majority of the points taken in the oil/IL sample boundary films, formed by the oil/IL blends, showed the presence of both boron and phosphorus. The presence of boron enriched patches and the reduced boron concentration seen by ^11^B NMR appears to confirm that boron in the boundary films originated from the IL “consumed” during the lubrication tests.

It should be remembered however, that physically adsorbed IL at the lubricated surface can also contribute to the observations. The mechanism of boron mobility toward the lubricated surfaces and its surface activity includes a combination of processes. Since lubrication of steel can result in a locally positive surface charge (exo-electron emission; Nakayama et al., 1992), anions will consequently be preferentially attracted to the surface. It has been demonstrated for the same OPAG/IL mixture that such an electro-diffusion process is indeed controlled by the surface charge (Hjalmarsson et al., under review). Thermomechanical stresses cause the anions to decompose and it is thought that decomposition starts with the cleavage of a B-O bond, which simulations demonstrate to be energetically most vulnerable (Golets et al., 2016). Boron may contribute to formation of B_2_O_3_ and BPO_4_ while phosphorus may contribute to formation of phosphates and polyphosphates (Sharma et al., 2016). These compounds are known to provide wear resistant boundary films (McFadden et al., 1997; Shah et al., 2013). Compared to the case of borate ester addition to the IL/oil blend (Sharma et al., 2016), the use of orthoborate anions provides more boron in the boundary films enhancing the anti-wear performance. FIB-EDS was used to probe the thickness and composition of the selected patches, particularly since interfacially bound, unreacted IL might contribute to the EDS spectra in Figures 7, 8.

**Figure 9:**
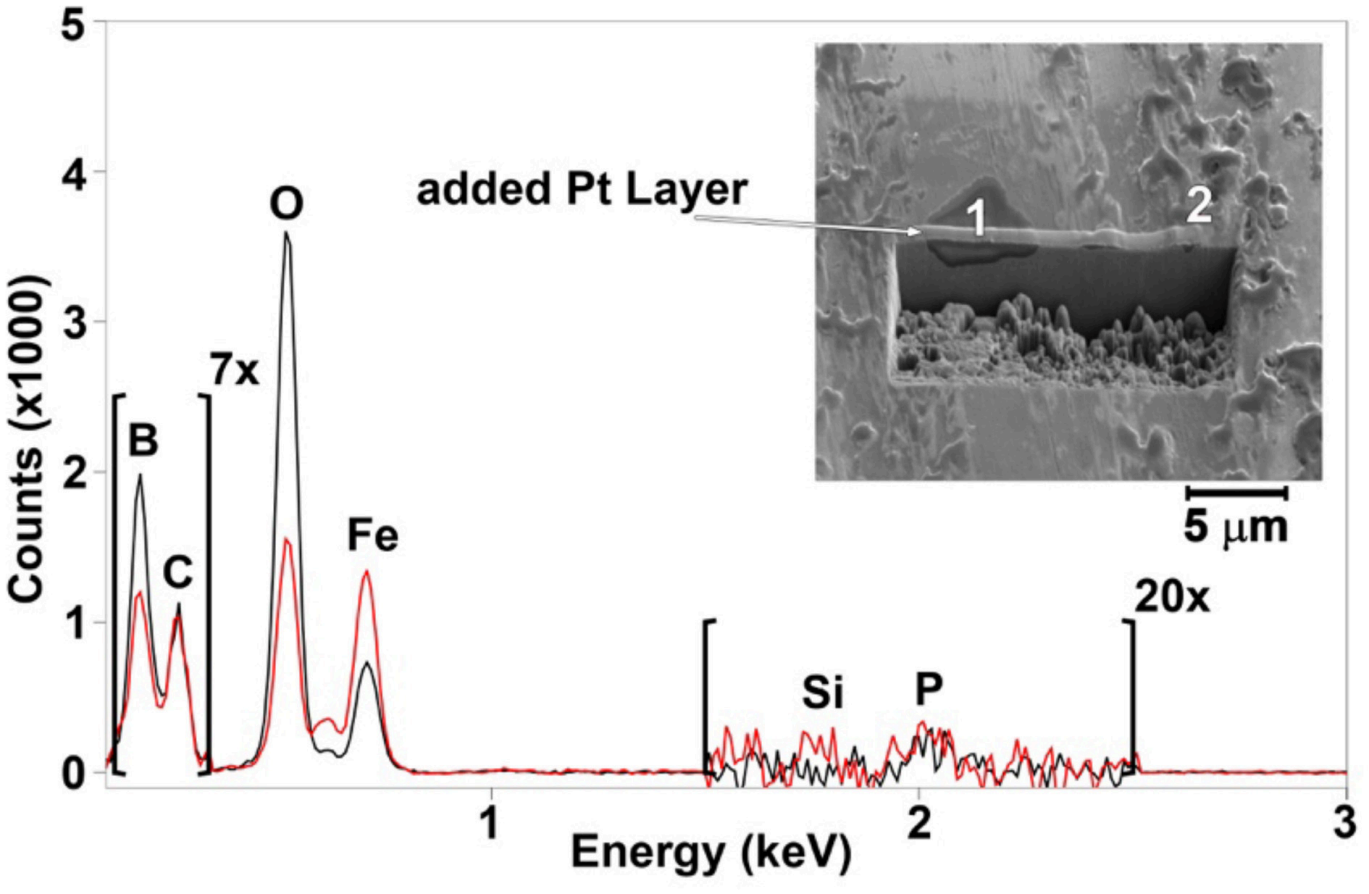
EDS spectra for points 1 (in black) and 2 (in red) and the corresponding FIB cross sectional cut of a patch formed on the surface lubricated by the ME/IL blend.

**Figure 10:**
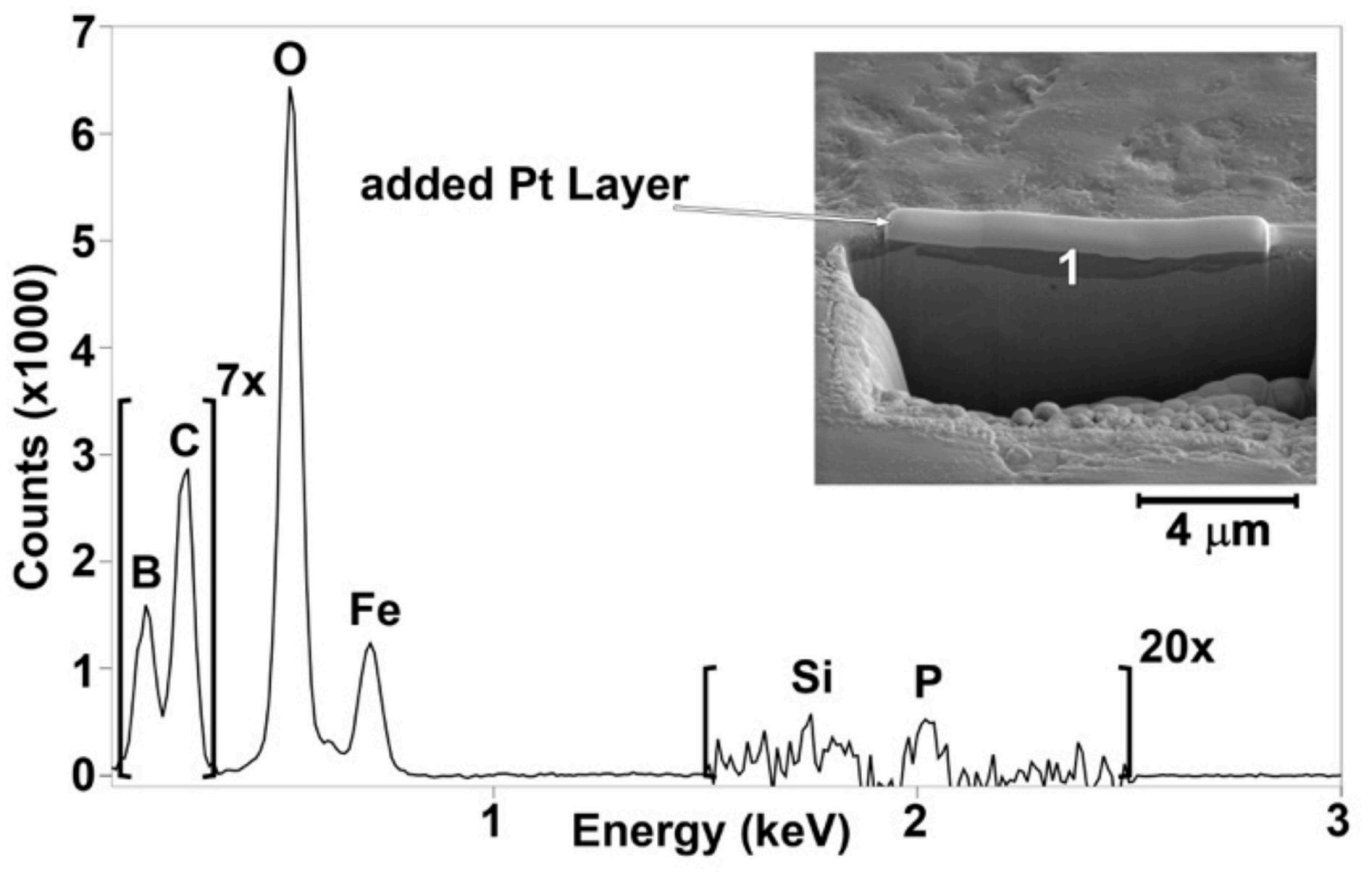
EDS spectrum for point 1 in the FIB cross sectional cut of a patch formed on the surface lubricated by the OPAG/IL blend.

Figure 9 shows results for the ME/IL blend. The boron containing patch is rather wide and thick. Judging by the peak intensity, the boron content is much higher than the phosphorus content. A cross sectional cut of the patch reveals a uniform structure of up to 1,600 nm in thickness, probably composed largely of iron oxides, B_2_O_3_ and HBO_2_-III (Sharma et al., 2016; Spadaro et al., 2018). Similar observations can be made for the patches formed by the OPAG/IL blend. Figure 10 illustrates that the patch is once again enriched in boron with a smaller amount of phosphorus. Furthermore, the cross section of the patch reveals a homogeneous structure which is similar to, but much thinner than (800 nm), that formed by the ME/IL blend. This agrees well with the boron consumption trends revealed by the ^11^B NMR results. Hence, it can be concluded that the protective boundary films are similar in nature regardless of the base oil in which the IL was dissolved.

## Conclusion

The choice of IL as “next generation additive” to polar oils appears to be well-justified. It is clear that this non-halogenated IL forms stable, wear-reducing, boundary films in polar biodegradable oils. Importantly, it is the bis(mandelato)borate anion, rather than (as generally assumed) the tetraalkylphosphonium cation, which appears to answer for the majority of the composition of the boundary film.

The wear reduction is dramatic compared to the unformulated base oils in both cases, but there is a very strong dependence on the oil type, at least at the single, relevant temperature used here. The monoester performs better as a lubricant than the polyalkylglycol in the absence of IL, but this behavior is essentially reversed when IL is used as additive. In both cases reactions occur and wear films are formed but the wear film is much thinner in the polyalkylglycol than in the monoester and much less IL is consumed.

The study shows that the fields of chemistry and tribology are inextricably linked and provides a clear path forward for the chemical design of IL based anti-wear additives in synergy with the biodegradable oil. The choice of anion is as important as that of the cation and should be matched to both the cation and the oil.

## Author Contributions

PR and BM performed and analyzed experiments. SG, OA, IF, and MR conceived the project. All authors contributed to the interpretation and writing of the manuscript.

### Conflict of Interest Statement

The authors declare that the research was conducted in the absence of any commercial or financial relationships that could be construed as a potential conflict of interest.
