# Supplementary material for: Non-halogenated Ionic Liquid Dramatically Enhances Tribological Performance of Biodegradable Oils
Source: Front Chem. 2019 Feb 28;7:98. doi: 10.3389/fchem.2019.00098 (PMC6413702; doi:10.3389/fchem.2019.00098)
Supplement: Supplementary file 1 [file Data_Sheet_1.pdf]

Electronic Supporting Information to the Article

Non-Halogenated Ionic Liquid Dramatically Enhances Tribological Performance of Biodegradable Oils

P. Rohlmann[a,†], B. Munavirov[a,b,†], I. Furó[b], O. N. Antzutkin[c], M. W. Rutland[b,d,] and S. Glavatskih[a,e]*

[a]Machine Design, KTH Royal Institute of Technology, SE-100 44 Stockholm, Sweden
[b]Department of Chemistry, KTH Royal Institute of Technology, SE-100 44 Stockholm, Sweden
[c]Chemistry of Interfaces, Luleå University of Technology, SE-971 87 Luleå, Sweden
[d]SP Technical Research Institute of Sweden, SP Chemistry, Materials and Surfaces, SE-114 86 Stockholm, Sweden
[e] Department of Electrical Energy, Metals, Mechanical Constructions & Systems, Ghent University, Ghent, 9000, Belgium

**Email: mark@kth.se*

Table of Content

1. Viscosity Data S1
2. Ball-on-three plates experimental setup S2
3. Friction Data S2
4. Wear scars S3
5. Maximum Hertzian Contact Pressure S3

1. Viscosity data

Table S1: Properties of the tested lubricants at atmospheric pressure

Fluid	Fluid Dynamic Viscosity [mPa s]	
	40°C	100°C
ME	3.1 ± 0.1	1.3 ± 0.2
ME / IL	3.5 ± 0.1	1.7 ± 0.2
OPAG	43.0 ± 0.2	7.7 ± 0.3
OPAG / IL	52.6 ± 0.2	9.2 ± 0.1

† These authors have contributed equally to this work

2. Ball-on-three plates experimental setup

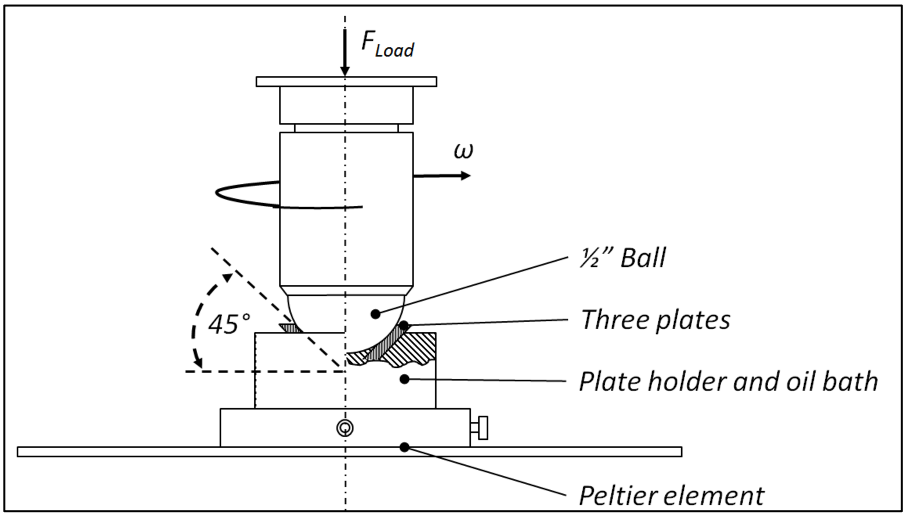

Figure S1: Ball-on-three-plates experimental setup

3. Friction data

Normal and friction forces were recorded with an acquisition frequency of 1 Hz. In the beginning of each test a running-in process resulted in a large variation of the coefficient of friction. The variation decreased with time in all cases but returned for the case ME + IL. The addition of IL to OPAG clearly reduced the friction compared to the neat oil.

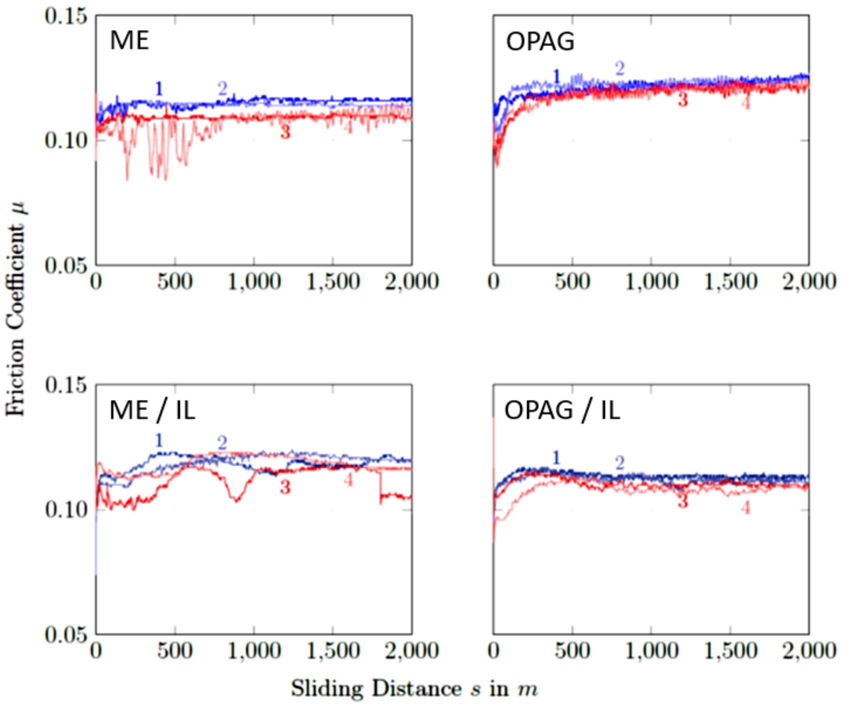

Figure S2: Coefficient of friction of oils and mixtures with IL at 90°C

4. Wear scars

A contact between the ball and a plate should produce a wear scar of a circular shape. However, the elliptical shape of the wear scars is due to the 45° inclination of the plates (see **Figure S1**), thermal expansion of the setup during the experiments and tolerances. This effect is more visible for the smaller wear scars.

5. Hertzian contact pressure

The maximum Hertzian contact pressure was calculated using Hertzian equation as below (Johnson, 1987):

$$P = 21.34\ N$$

Equivalent modulus of elasticity:

$$\frac{1}{E^*} = \frac{1-{\nu_1}^2}{E_1} + \frac{1-{\nu_2}^2}{E_2}\ , \qquad (1)$$

$$\nu_1 = \nu_2 = 0.3$$
$$E_1 = E_2 = 210\ GPa$$

Equivalent radius:

$$\frac{1}{R} = \frac{1}{R_1} + \frac{1}{R_2}\ , \qquad (2)$$

$$R_1 = 6.35\ mm$$
$$R_2 = \infty$$

Maximum Hertzian contact pressure:

$$p_{max} = \sqrt[3]{\frac{6\ P\ {E^*}^2}{\pi^3\ R^2}} = 1.11 GPa \qquad (3)$$
